# Early Experience in the Treatment of Intra-Cranial Aneurysms by Endovascular Flow Diversion: A Multicentre Prospective Study

**DOI:** 10.1371/journal.pone.0012492

**Published:** 2010-09-02

**Authors:** James V. Byrne, Radu Beltechi, Julia A. Yarnold, Jacqueline Birks, Mudassar Kamran

**Affiliations:** 1 Nuffield Department of Surgical Sciences, University of Oxford, Oxford, United Kingdom; 2 Centre for Statistics in Medicine, University of Oxford, Oxford, United Kingdom; Hungarian Academy of Sciences, Hungary

## Abstract

**Introduction:**

Flow diversion is a new approach to the endovascular treatment of intracranial aneurysms which uses a high density mesh stent to induce sac thrombosis. These devices have been designed for the treatment of complex shaped and large size aneurysms. So far published safety and efficacy data on this approach is sparse.

**Material and Methods:**

Over 8 months, standardized clinical and angiographic data were collected on 70 patients treated with a flow diverter device (SILK flow diverter (SFD)) in 18 centres worldwide. Treatment and early follow up details were audited centrally. SFDs were deployed alone in 57 (81%) or with endosaccular coils in 10 (14%) aneurysms, which included: 44 (63%) saccular, 26 (37%) fusiform shapes and 18 (26%) small, 37 (53%) large, 15 (21%) giant sizes. Treatment outcome data up to 30 days were reported for all patients, with clinical (50 patients) and imaging (49 patients) follow up (median 119 days) data available.

**Results:**

Difficulties in SFD deployment were reported in 15 (21%) and parent artery thrombosis in 8 (11%) procedures. Procedural complications caused stroke in 1 and serious extracranial bleeding in 3 patients; 2 of whom developed fatal pneumonias. Delayed worsening of symptoms occurred in 5 patients (3 transient, 1 permanent neurological deficit, and 1 death) and fatal aneurysm bleeding in 1 patient. Overall permanent morbidity rates were 2 (4%) and mortality 4 (8%). Statistical analysis revealed no significant association between complications and variables related to treated aneurysm morphology or rupture status.

**Conclusion:**

This series is the largest reporting outcome of the new treatment approach and provides data for future study design. Procedural difficulties in SFD deployment were frequent and anti-thrombosis prophylaxis appears to reduce the resulting clinical sequelae, but at the cost of morbidity due to extracranial bleeding. Delayed morbidity appears to be a consequence of the new approach and warrants care in selecting patients for treatment and future larger studies.

## Introduction

Coil embolisation has proved an effective treatment for most intracranial aneurysms but larger aneurysms, fusiform shaped aneurysms, and those with wide necks are technically challenging. Such aneurysms are also more liable to recurrence which occurs in 20–30% [1] and results in 10–12% of patients requiring retreatment [2]–[3]. Adjuvant stenting was proposed in the mid-1990's, initially for fusiform aneurysms [4] and more recently to support the neck region of intracranial saccular aneurysms and to prevent recurrence [5]. The principle that a stent placed in the parent artery can reduce blood flow in the sac of an aneurysm to the point of stagnation and thrombosis has been exploited for sometime in other anatomies and has now been extended to the intracranial vasculature in a new range of implants designed to be sufficiently flexible for intracranial navigation.

These new devices, termed flow diverters, recently became available for clinical use. The SILK stent (Balt Extrusion, Montmorency, France) was approved in 2007. This self-expanding flexible stent is constructed of woven nitinol strands with low porosity, to retain blood flow in the parent artery and exclude the aneurysm sac. In order to assess the safety and efficacy of the SILK flow diverter (SFD), a multicentre registry was established. The intention of this study is to assess the device's performance in routine clinical practice by collecting data from as many users as possible.

## Materials and Methods

### Methods

#### a). Patient recruitment

All centres using the SFD during 8 months (March to November, 2008) were invited to send anonymised and coded data to the Oxford Neurovascular and Neuroradiology Research Unit (ONNRU). Thirty centres world-wide used the device during this period. Eighteen participated and returned reports of consecutive treatments using a standard case report form (CRF). The study protocol defining data to be collected was established prior to recruitment and prior to most invitations.

**Figure 1:**
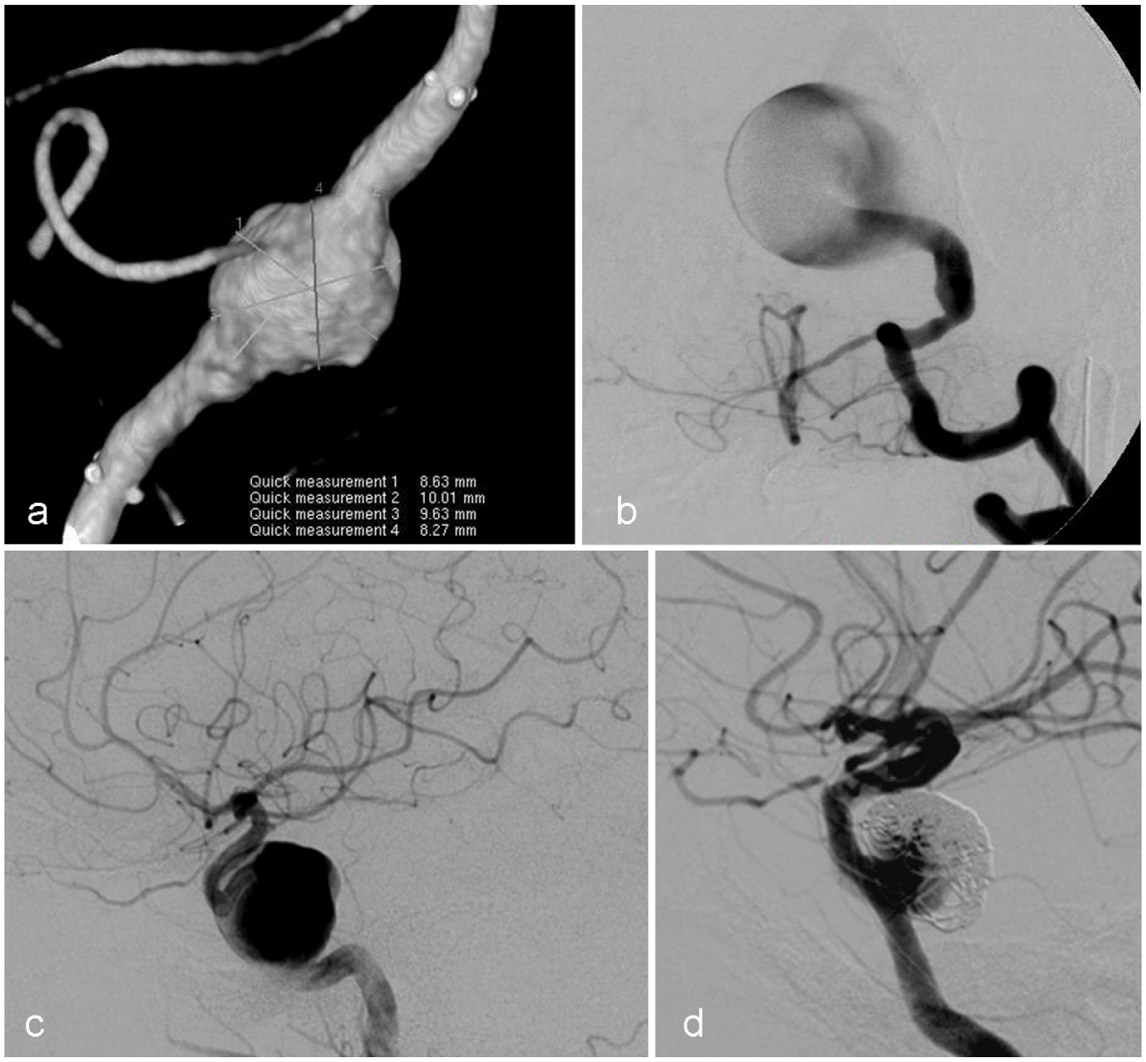
Representative angiograms of aneurysms treated with the SFD. Fusiform vertebral artery aneurysm (a), giant saccular aneurysm at the vertebro-basilar junction (b), large cavernous carotid artery saccular aneurysm (c) and a recurrent cavernous carotid artery aneurysm (d).

Patients were selected for treatment locally at the treating centre on the basis that the target aneurysm was unsuitable for conventional endovascular or neurosurgical treatments (see Figure 1). No details of the patients' age and gender were collected to maintain absolute anonymity. The following exclusion criteria were set by the study Steering Committee: any contraindication to antiplatelet drugs, pregnancy, breast feeding, and aneurysms considered treatable with coils alone. The study protocol was defined as clinical audit using the UK National Research Ethics Service guidance (Defining Research 2007) and did not require research ethics committee approval. Each centre was responsible for obtaining appropriate permissions for data sharing.

#### b). Data collection

Prior to treatment, modified Rankin scores (mRS) and Glasgow Coma Scores (GCS) were recorded. Therapists were asked to record any untoward procedural events (UPE) during procedures, any acute clinical complication and a GCS after treatment. The CRF described UPEs as; catheterization failure, poor SFD positioning, SFD migration, poor SFD opening on deployment, haemorrhage during or after the procedure, complications related to other devices, and partial or complete thrombosis of the parent artery.

A standard description of the aneurysm to be treated was made. These details included: rupture status, location, saccular or fusiform shape, and sac dimensions (neck width, maximum diameter, and dome to neck length). Details of prophylactic anti-thrombosis therapies were reported. These included: the dose and type of drugs used, and the duration of prescriptions.

#### c). Follow-up protocols

A standard report of each patient's clinical status was requested at the time of follow-up imaging. In order to assess the effectiveness of the SFD at inducing thrombosis, it was requested that follow-up catheter angiography was performed 1 month after stopping antiplatelet therapy. The timing of interval imaging was therefore left to the centres. The follow-up period range was 9–528 days (median 119 days). Assessments of the degree of aneurysm thrombosis after SFD placement were rated as complete occlusion (OG1), neck remnant (OG2) and residual sac filling (OG3), at end-of-treatment and follow-up [2].

#### d). Statistical analysis

Potential associations between the procedural and delayed complications and the variables related to aneurysm morphology, presentation, and use of adjuvant coils were explored using Fisher's exact test (aneurysm shape, use of coils, location, and previously ruptured vs unruptured aneurysms) and the Pearson's chi-square test (sac size).

### Materials

#### a). Patients

Seventy patients were entered in the study. Patients' prior degrees of disability and symptoms due to the aneurysms were: mRS = 0 in 30, mRS = 1 in 19, mRS = 2 in 12, mRS = 3 in 2, mRS = 4 in 6 and mRS = 5 in 1 patients. Thus 30(43%) treatments were performed for patients without symptoms, 31(44%) with symptoms but no or only mild disability and 9(13%) with moderate or significant disablity i.e. mRS 3–5.

#### b). Aneurysms and SFD Sizing

Sixty target aneurysms were unruptured and 4 of the 10 ruptured aneurysms were treated within 30 days of haemorrhage. They comprised 44(63%) saccular and 26(37%) fusiform shapes. There were 15(21%) giant (>25 mm), 37 (53%) large (10–25 mm) and 18 (26%) small (<10 mm) aneurysms. Details of target aneurysms are presented in Table 1. Multiple aneurysms were identified in 5 patients (4 with 2 and 1 with 3 aneurysms). One patient with 2 aneurysms had both treated with the SFD in separate procedures (without complication) and another patient was treated for a co-incidental aneurysm with coils alone at the same procedure. These treatments have not been included in the analysis of primary treatment outcomes. The remaining co-incidental aneurysms were not treated during the study period.

**Table 1:** Aneurysm locations presented as maximum sac size, neck width, and morphology.

	Saccular aneurysms (n = 44)						Fusiform (n = 26)			Total (n = )
Location of the aneurysm	Maximum dimension (mm)			Neck width (mm)*			Maximum dimension (mm)			
	<10	10–25	>25	<4	4–10	>10	<10	10–25	>25	
ICA cavernous	1	6	2	1	5	3	2	7^†^	1	19
COA	5	8	3	2	12	2	2	1	0	19
PCoA	1	4	1	1	4	1	0	0	0	6
MCA	1	3	0	0	3	1	0	1	1	6
BA trunk	0	2	0	0	1	1	1	1^†^	1	5
SCA	0	1	0	0	1	0	0	0	0	1
AICA	0	1	1	0	2	0	0	0	0	2
PICA	1	0	0	0	1	0	0	1‡	0	2
VA	2‡	1*,†	0	1	1	0	2	0	5‡	10
Total (n = )	11	26	7	5	30	8	7	11	8	70

Abbreviations: n (numbers of aneurysms), mm (millimetres), AICA (anterior inferior cerebellar artery), BA (basilar artery), COA (carotid ophthalmic artery), ICA (internal carotid artery), MCA (middle cerebral artery), PCoA (posterior communicating artery), PICA (posterior inferior cerebellar artery), VA (vertebral artery).

*For one saccular aneurysm the neck width was not measured; †Procedure abandoned (failed treatments);

‡ Second procedures.

#### c). Centres

The number of treatments performed in contributing centres varied from 1–9. Treatments per centre were: 1 in 2, 2 in 6, 3 in 2, 4 in 1, 5 in 3, 7 in 2, 8 in 1 and 9 in 1. Thus half the centres performed only 1–3 procedures and for most these were their first using the SFD.

#### d). SFD Deployment Technique

Sixty treatments were performed with SFD alone and 10(14%) with adjuvant endosaccular coils. Additional coils were used for parent vessel occlusion during 1 treatment. During the study period many centres were supported by a proctor introduced by the manufacturer and treatments were performed under general anaesthesia according to the current instructions-for-use of the SFD. Briefly, the technique involves positioning a delivery microcatheter (Vasco 21, Balt, Montmorency, France) with its tip distal to the aneurysm and then pushing the SFD, which is applied to a delivery microwire, to the tip of the delivery microcatheter. The system is then aligned with the aneurysm under x-ray fluoroscopy and the SFD deployed by unsheathing it from the constraint of the microcatheter. This involves a combination of pushing the delivery wire and retrieving the microcatheter to allow the SFD to expand and to compensate for any resulting fore-shortening. The SFD can be retrieved into the microcatheter and removed or repositioned when less than 80% of its length has been extruded. No retrieval is possible thereafter.

#### e). Antiplatelet regimens

All centres prescribed heparin and antiplatelets during treatments. Antiplatelet prescriptions in the majority of patients were a combination of aspirin and clopidigrel (46 pre-treatment and 49 post-treatment) or aspirin and dipyridamole (19 pre-treatment and 11 post-treatment). Single therapy with aspirin (2 pre-treatment and 3 post-treatment) or clopidigrel (3 pre-treatment and 7 post-treatment) was used in a minority. Reports of post-treatment prescriptions were incomplete. Reported intervals to stopping clopidigrel or dipyridamole after treatments were: after 2 months in 5 patients, after 3 months in 3 patients, after 4 months in 17 patients, after 5 months in 5 patients, and after 6 months in 9 patients.

## Results

### a). Procedures

Sixty seven (96%) primary treatments were completed. In 3(4%) treatments, an SFD could not be deployed for technical reasons and the procedures were abandoned. These were: failure to catheterise the aneurysm bearing artery, inability to open the SFD correctly and failure to advance a long SFD through the delivery catheter. In the last case, the patient was successfully treated with two shorter SFDs after the study period ended. Another 3(4%) patients required repeat procedures because of suboptimal SDF deployment. In 2, the initial SFD shortened and another SFD was placed at a second procedure without complication. In the third, positioning 2 SFDs in a dolichectatic artery failed due to foreshortening and a second procedure was performed a few days later with an additional LEO stent (Balt Extrusion). Arterial patency was confirmed after 48 hours but the patient died from pneumonia 1 month later.

### b). Untoward Procedural Events (UPEs)

One or more UPEs were reported in 20 patients. They were: 12(17%) poor SFD opening on deployment, 7(10%) partial or complete parent artery thrombosis (PAT), 6(8%) poor SFD positioning, 4(6%) SFD migration, 3(4%) post procedure extracranial haemorrhages and 1(1.5%) complication related to another device. No intracranial haemorrhage was reported.

Thus, the commonest UPE was poor SFD opening on deployment. In 4 procedures, poor SFD positioning and in 3 procedures SFD migrations were concurrently reported. Thus overall in 15(21%) of procedures SFD deployment difficulties (DD) were reported and amalgamated for statistical analysis. The consequences of these events were that 3 patients required repeat procedures, as described above. In 1 other patient, the operator decided to occlude the parent artery with coils because of incomplete SFD opening. This was without clinical worsening.

The 7 reports of PAT occurred together with reports of SFD DD in 6 procedures (poor opening in 3, positioning in 1 and both in 2). At the remaining procedure, branch artery thrombosis was treated successfully with abciximab. Subsequent extracranial bleeding complicated 3 procedures. In 1 patient this occurred after endovascular access via the cervical carotid artery. A resulting neck haematoma was so serious that the patient required a week of intensive care treatment and the relevant internal carotid artery was occluded on follow-up angiography. A significant acute groin haematoma was reported in 1 further patient and 1 patient developed serious gastric bleeding 48 hours post-procedure and died of pneumonia 2 weeks later.

### c). Procedural Morbidity

The GCS of patients immediately prior to treatment were: GCS 15 n = 61 (plus 2 abandoned), GCS 10–14 n = 5 (plus 1 abandoned) and GCS<10 n = 1. In the 48 hours after treatment, 1 patient worsened (GCS 13 to 5) and 2 patients improved (GCS 14 to 15). Worsening followed a thromboembolic complication, which caused a new permanent hemiparesis. This was attributed to thromboembolism caused by adjuvant endosaccular coils. Post-procedure MRI showed a new infarct in the temporal lobe and delayed angiography showed the SFD to be patent. There were no procedural deaths reported in the acute period. However, 2 patients whose treatments were complicated by UPEs, died of pneumonia within 1 month of the procedure. Thus the immediate procedural related morbidity was 1 new neurological deficit and 2 deaths.

### d). Delayed Morbidity

**Figure 2:**
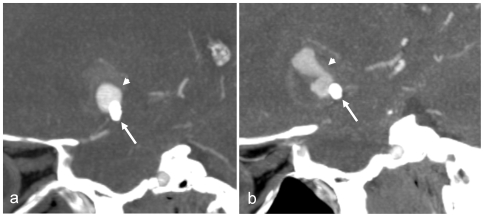
Partially thrombosed aneurysm after treatment with the flow diverter. CT angiograms showing a residual lumen within a large partially thrombosed fusiform aneurysm of the middle cerebral artery. Follow-up CTA (a) was performed 4 months and (b) 6 months after SFD (arrows) placement. The second follow-up study shows enlargement of the residual aneurysm lumen (arrow heads) and was performed after a new haemorrhage (not shown).

Follow-up reports on clinical outcomes were returned on 50(71%) patients. Delayed worsening of symptoms and/or new abnormal neurological signs were reported in 6(12%) of these 50 patients. Transient events in 3 patients occurred within 30 days of treatment and were attributed to increased mass effect following aneurysm thrombosis. This caused exacerbation of headaches and in 2 patients (with aneurysms of the cavernous carotid artery) worsening opthalmoplegia. Permanent deficits developed in 3 patients and were attributed to delayed thrombosis of the SFD in the first patient (who developed hemiparesis 10 days post-procedure), increased mass effect in the second (who developed a new facial palsy a few days after treatment of a giant basilar artery aneurysm and died 2 weeks later due to increased brain stem compression) and delayed aneurysm bleeding in the third patient. In this last case, follow-up angiography at 5 months showed residual aneurysm filling. Two months later the patient experienced increasing headache and acute hemiparesis. CT showed aneurysm enlargement and acute haemorrhage (Figure 2). The patient died 4 weeks later despite surgical by-pass. He had required long-term anticoagulation with warfarin because of a prosthetic heart valve and remained on aspirin after clopidigrel was stopped 2 months following SFD deployment.

Thus the overall morbidity reported amongst 50 patients with follow-up was 2 permanent neurological deficits and 4 deaths, i.e. morbidity 2(4%) and mortality 4(8%). All clinical complications are summarized in Table 2.

**Table 2:** Clinical complications observed in patients treated with SFD.

Aneurysm morphology			Complications	Cause or contributing events	Outcome
Location	Size (mm)	Shape			
			***Procedure related:***		
BA	13	S	Cerebral infarction	Thromboembolism attributed to the use of coils	Hemiparesis
BA	32	F	Pneumonia	Patient required second procedure due to poor SFD positioning	Died
PcomA	32	S	Gastric bleeding followed byPneumonia	Antiplatelet drugs	Died
CCA	35	F	Neck hematoma	Carotid puncture for EVT and antiplatelets drugs	ProlongedICU stay
MCA	30	F	Groin bleeding	Antiplatelet drugs	Resolved
			***Delayed complications:***		
AICA	11	S	Worsening of brainstem compression symptoms	Aneurysm thrombosis and mass effect	Resolved
CCA	27	F	Worsening of cranial nerve palsy	Aneurysm thrombosis and mass effect	Resolved
COA	22	S	Worsening of cranial nerve palsy	Aneurysm thrombosis and mass effect	Resolved
CCA	10	S	Parent artery occlusion	Poor SFD deployment	Hemiparesis
BA	35	F	New cranial nerve palsy	Aneurysm thrombosis and mass effect	Died
MCA	24	F	Late increase in aneurysm size and bleeding	Uncertain; possible use of warfarin anti coagulation	Died

Abbreviations: BA (basilar artery), PcomA (posterior communicating artery), CCA (carotid cavernous artery), MCA (middle cerebral artery), AICA (anterior inferior cerebellar artery), COA (carotid ophthalmic artery), S (saccular), F (fusiform), EVT (endovascular therapy).

### e). Angiographic outcomes

The end-of-treatment (EOT) assessments of the degree of aneurysm occlusion for 68 treated aneurysms (67 patients) were: OG1 in 7(10%), OG2 in 4(6%) and OG3 in 57(84%) aneurysms. Reports of follow-up angiograms were returned for 49(72%) aneurysms. The degrees of aneurysm occlusion on follow-up were: OG1 in 24(49%), OG2 in 13(26%) and OG3 in 12(25%). Thus all but 2 aneurysms were unchanged or improved on follow up (1 OG1 and 1 OG2 dropped to OG3 on follow up) (Table 3).

**Table 3:** Angiographic outcomes for the aneurysms treated with SFD.

Occlusion grade	Angiographic status at EOT	Follow up available	Occlusion status of aneurysms at follow up relative to EOT	Angiographic status at follow up	Intervals (weeks) since treatment			
					0–20	20–40	40–60	60–80
OG1	7	5	OG1, 4; OG2, 0; OG3,1	24	17	3	2	2
OG2	4	4	OG1, 2; OG2, 1; OG3,1	13	13	0	0	0
OG3	57	40	OG1, 18; OG2, 12; OG3,10	12	9	3	0	0
OG1%	10%	10%		49%	44%	50%	100%	100%
95% CI	3–17%	3–17%		35–63%	28–60%	11–89%	-	-

Angiographic outcomes for the cohort of aneurysms with complete follow up i.e. end of treatment and follow up angiogram.

Abbreviations: OG1 (complete occlusion); OG2 (neck remnant); OG3 (saccular filling).

Follow up imaging showed parent artery occlusion (PAO) in 7(14%) and a degree of arterial narrowing in 3(6%). Three PAOs were associated with UPEs (neck haematoma, DD, and deliberate PAO with coils), 1 was attributed to non-compliancy with antiplatelet treatment and 3 occurred for no apparent reason. The degree of arterial narrowing reported in 3 cases was not graded but reported as severe in 1 patient. In 2 cases of arterial stenosis on follow up imaging, UPEs had occurred during treatments (poor SDF opening and thrombosis requiring salvage abciximab).

**Figure 3:**
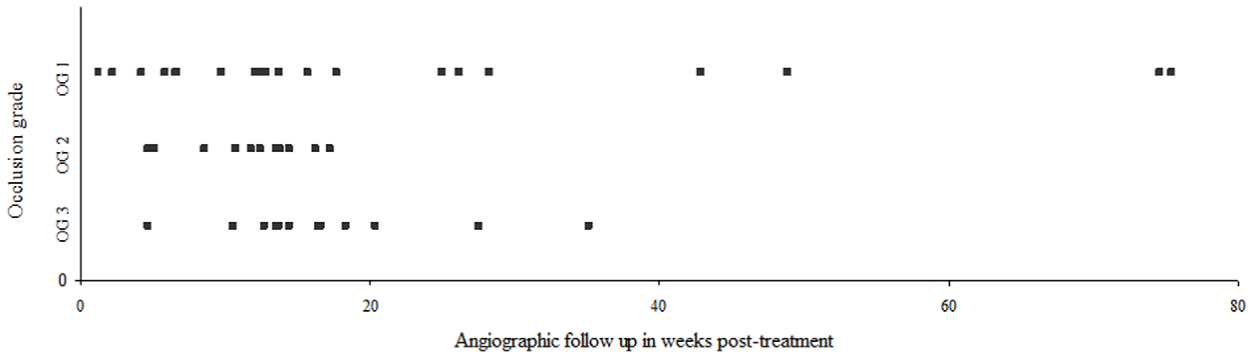
Timings and results of angiographic follow up. Plot of angiographic outcomes against follow up times in weeks.

The frequency of complete occlusion (i.e. OG1) and a possible effect of the timing of follow-up imaging was investigated by dividing the cohort at 20 week intervals post-treatment and comparing the within-group percentage occlusion grades. This analysis showed that the proportion of cured aneurysms increased with time intervals (Table 3 and Figure 3).

### f). Statistical Analysis

No statistically significant relationship was found between the variables related to aneurysm morphology, rupture status, use of adjuvant coils and the occurrence of procedural and delayed complications (Table 4).

**Table 4:** Effect of patient and procedure related variables on SFD deployment difficulty, flow disturbance, and delayed neurological complications.

Procedural and patient variables		Deployment difficulty		Flow disturbance		Delayed neurological complications	
		Present	Absent	Present	Absent	Present	Absent
**Sac morphology**	Saccular	9	36	5	40	4	29
	Fusiform	6	19	2	23	2	16
		*p-value 0.76*		*p-value 1.0*		*p-value 1.0*	
**Aneurysm size**	Small	4	18	2	20	3	13
	Large	7	30	3	34	2	24
	Giant	4	7	2	9	1	8
		*p-value 0.42*		*p-value 0.61*		*p-value 0.56*	
**Coils**	Used	4	5	1	8	2	5
	Not used	11	50	6	55	4	40
		*p-value 0.09*		*p-value 1.0*		*p-value 0.18*	
**Location**	Anterior	12	36	5	43	4	31
	Posterior	3	19	2	21	2	14
		*p-value 0.36*		*p-value 1.0*		*p-value 1.0*	

Untoward events reported during and after treatments related to aneurysm shapes, sizes location, and use of coils.

Deployment difficulty  =  poor opening, poor positioning, or migration of SFD.

Flow disturbance  =  partial or complete thrombosis of parent artery.

## Discussion

The concept of a stent capable of inducing thrombosis of intracranial aneurysms has stimulated the development of flow diverters over the last 15 years. Several researchers have demonstrated their potential to disrupt endosaccular blood flow in experimental aneurysms, and systematic haemodynamic studies have informed their design [6]. These established 70% porosity (defined as the proportion of open area to total area of the stent) as the optimum [7]. Lower porosity covered stents have generally proved too inflexible for intracranial use and results of stent-alone treatments using conventional high porosity stents have been inconsistent [8]. Some operators have proposed the use of double or overlapping conventional stents to increase the effective stent porosity [9] and new covered devices are being tested [10].

Against this background, flow diverters designed with low porosity and high flexibility were first used in humans in late 2006. To date, the few reports of their efficacy have been encouraging. Lylyk et al. [11] reported a series of 53 patients treated in Buenos Aires using the Pipeline (Chestnut Medical Technologies, Inc., Menlo Park, CA). The aneurysm types were predominantly saccular with only 12% fusiform and nearly half were small in size. Clinical experience with the same flow diverter was collected on 31 patients treated in the “Pipeline Embolization Device in the Intracranial Treatment of Aneurysms (PITA) Trial” conducted between January and November 2007 [12]. One of the 4 contributing centres recently reported on 8 additional aneurysms [13]. Few procedure-related neurological complications were reported; a new stroke rate of 6% in the PITA trial and none in the Buenos Aires report. The latter reported delayed exacerbation of aneurysm related cranial nerve palsies in 3 patients.

Complication rates reported in this study were similar with only one acute new neurological deficit despite technical difficulties associated with SFD deployment in 21% of procedures. Like the Buenos Aires experience, a minority of our patients developed delayed neurological symptoms. The cause of transient worsening of symptoms after induced aneurysm thrombosis is generally attributed to aneurysm expansion. However, the low porosity of flow diverters theoretically increases their likelihood of occluding covered side branch arteries which might also cause worsening of symptoms [13]. In this registry, partial or complete parent artery thrombosis affected 7 procedures and was attributed to the SFD deployment difficulties rather than inadequate antithrombotic prophylaxes since these were given during all the procedures. However, in 1 case it was caused by the device covering a large branch artery. A cause of 1 delayed complication was parent artery occlusion found on follow-up imaging. The relative contribution of aneurysm expansion or branch artery occlusion in patients developing new neurological deficits is therefore difficult to assess. Our experience suggests a real vulnerability of parent artery blood flow after SFD deployment, perhaps due to a combination of its relatively low radial force and high metal density, which high dose antiplatelet therapy mitigates.

Treatment using SFDs after acute SAH raises several concerns. Firstly, after SAH there is a natural reluctance to prescribe antiplatelets prior to securing the aneurysm against rebleeding. In the minority of patients treated after acute SAH in this study, it was the usual practice to withhold prophylaxis with antiplatelet treatment until the SFD was deployed; accepting a small additional risk of thrombotic complications. The second issue is the longer delay to complete thrombosis of the aneurysm after SFD treatment than after clipping or coiling. During this period, the patient is at risk of more prolonged bleeding, should the aneurysm re-rupture, because of antiplatelet medication. One solution is to additionally pack the aneurysm sack with coils. In this cohort, adjuvant coils were only used in 1 of the 3 saccular aneurysms treated after acute SAH. Thus, when it proves difficult or impossible to place coils, treating physician have to assume that the alteration of local haemodynamics induced by the SFD reduces the risk of rebleeding. Currently, there is insufficient experience to prove this assumption.

The relationship between complications and anti-thrombotic prophylaxis was clear in the patients who suffered haemorrhagic complications but its contribution to delayed events is complex. In this cohort, patient non-compliance caused delayed spontaneous artery occlusion and flow disturbances observed during treatments would probably have resulted in more frequent thrombotic events without the use of heparin or antiplatelets. Its contribution to the subsequent evolution of endosaccular thrombosis is unclear and needs further systematic study. It is tempting to attribute concurrent long-term anticoagulation and aspirin therapy as the cause of delayed aneurysm enlargement and haemorrhage, seen in one patient. On the same basis, antiplatelet therapy might contribute to symptomatic worsening attributed to sac swelling observed in the early post-procedure period. Another unanswered question raised by our results, is whether it is a factor in the asymptomatic failure of sac occlusion, seen on follow up imaging, in a minority of aneurysms.

Limitations to this study are incomplete follow-up imaging, no longitudinal data and the relatively short follow up period. The 50% rate for complete occlusion up to 3 months is similar to the Buenos Aires experience [11]. Their longitudinal follow-up suggested that induced thrombosis was progressive, with the 3-month 56% rate of complete occlusion rising to 95% at 12 months [11]. Progressive improvement in occlusion rates were reported by Zentano et al. [14] and no recurrence was reported for Pipeline treated aneurysms [13]. Our findings are similar but because our data are based on a single follow up time point, between-patients variations cannot be differentiated from variations over time. The subsequent behaviour of those aneurysms followed up early is unknown. The aneurysms reported here comprised a heterogeneous group, with a higher proportion of fusiform type and posterior circulation aneurysms than the series of Lylyk et al. or Szikora et al. [11], [13]. A concern is two aneurysms whose occlusion grades dropped relative to the EOT grade and a large study of sufficient numbers will be needed to show the long-term stability of thrombosis of different aneurysm types. This concern has resulted in a recent change in the manufacturer's instructions for use and the SFD should now only be used to support embolisation with coils.

The conclusions that can be drawn from the results of this study need to be considered with reference to its objectives and methodology. The intention was to collect data on the use of the SFD during a period when it was being first used in a large number of hospitals, by experienced practitioners who had either not previously used the device or had only done so in very few patients.

The centres contributed data on a voluntary basis and deserve our thanks. A registry can highlight early technical and clinical problems which would take much longer to identify without amalgamated data. Complete procedure reports allowed early recognition of deployment problems leading to a redesign of the SFD and an alteration in its instructions for use. The associations tested, though not statistically significant, should help to inform the design of future studies and devices. Several technical improvements have since been made to improve the SFD's visibility on x-ray fluoroscopy and deployment control. A new guide pusher has now been developed in addition to a new radio-opaque marker, which is more visible than the original version. However, conclusions about long-term efficacy must be tentative, because a possible bias to under reporting of poor outcomes cannot be excluded, without complete follow-up data. Further studies are needed to define the place of flow diverters in the endovascular treatment of aneurysms and it is too early to predict their ultimate role.
